# Biotreatment of pyrene and Cr(VI) combined water pollution by mixed bacteria

**DOI:** 10.1038/s41598-020-80053-2

**Published:** 2021-01-08

**Authors:** Shimei Ge, Junxia Gu, Wenjing Ai, Xinjiao Dong

**Affiliations:** College of Life and Environmental Science, Wenzhou University, Wenzhou, 325035 Zhejiang China

**Keywords:** Bacterial techniques and applications, Environmental impact

## Abstract

Pyrene and chromium (Cr(VI)) are persistent pollutants and cause serious environmental problems because they are toxic to organisms and difficult to remediate. The toxicity of pyrene and Cr(VI) to three crops (cotton, soybean and maize) was confirmed by the significant decrease in root and shoot biomass during growth in pyrene/Cr(VI) contaminated hydroponic solution. Two bacterial strains capable of simultaneous pyrene biodegradation and Cr(VI) reduction were isolated and identified as *Serratia* sp. and *Arthrobacter* sp. A mixture of the isolated strains at a ratio of 1:1 was more efficient for biotreatment of pyrene and Cr(VI) than either strain alone; the mixture effectively carried out bioremediation of contaminated water in a hydroponic system mainly through pyrene biodegradation and Cr(VI) reduction. Application of these isolates shows potential for practical microbial remediation of pyrene and Cr(VI) combined water pollution.

Pyrene has four fused benzene rings and belongs to the family of polycyclic aromatic hydrocarbons (PAHs), which are ubiquitous environmental pollutants that pose a hazard to aquatic organisms^1^. PAHs can be easily produced through the incomplete combustion of organic carbon-based substances frequently used in industry, transportation and other anthropogenic activities^2,3^. Pyrene is a typical PAH because it is difficult to degrade and can be easily transformed into benzo[a]pyrene (BaP), which is highly carcinogenic. Chromium (Cr), a heavy metal, is a persistent environmental pollutant that is widely used in various kinds of factories, such as textile dyeing, electroplating and wood preservation^4^. Cr(VI) and Cr(III) are the two main valent forms, but Cr(VI) is highly teratogenic, mutagenic and carcinogenic which results from the generation of reactive oxygen species and non-biodegradability and high solubility, is far more toxic than Cr(III) and frequently exists in untreated industrial effluents^4,5^.

Cr(VI) and pyrene are frequently discharged to the environment successively or simultaneously because of their various production pathways; Cr(VI) and pyrene pollution has caused severe environmental problems and influenced the normal growth of different plant species. Zhang et al.^6^ showed that PAHs, including pyrene and phenanthrene, had different effects on the growth of emergent wetland plants in hydroponics and soils, increasing or decreasing the growth depending on the specific species and pollutant concentrations. Cr(VI) with a concentration range of 75–100 μmol/L significantly decreased the root and shoot biomass of six peanut cultivars, which showed that Cr(VI) within this concentration range inhibited growth and was toxic to peanut^7^. Combined pollution generally has higher toxicity and is more difficult to deal with than either component alone because of the interaction between contaminants. For example, the joint toxicity of Cr(VI) and BaP had a significant antagonistic effect on the germination rate of *Lolium perenne*, although BaP in the concentration range of 1–4 mg/L accelerated the germination rate^8^. Thus, combined Cr(VI) and pyrene pollution was explored here, although some investigations of either component alone have been reported^9,10^.

There has been considerable research conducted on combined contaminants, such as Cr(VI) and phenanthrene or phenol^11,12^, or pyrene and other pollutants^13,14^. However, there has been limited research on Cr(VI) and pyrene combined pollution, apart from that of Wang^15^, which was focused on remediation of soil contaminated by Cr(VI) and pyrene using biochar alone or in combination with a bacterial consortium. There has been no published study on the bioremediation of water contaminated with combined Cr(VI) and pyrene to date. The aim of this study was to investigate biotreatment of Cr(VI) and pyrene combined water pollution by bacteria to decrease the toxicity of contaminated water on crops grown under hydroponic conditions, and optimize the application of inoculant bacteria for bioremediation of Cr(VI) and pyrene combined water pollution.

## Results

### Isolation and identification of simultaneous pyrene-degrading and Cr(VI)-removal bacteria

Bacterial strains were isolated that could grow in MM with pyrene and Cr(VI) and were tested for their ability to degrade pyrene and remove Cr(VI). Two strains, S41 and J122, which had such abilities, were selected for further analysis. 16S rRNA gene sequence analysis indicated that the two strains belonged to *Serratia* (GenBank No. MK027123) and *Arthrobacter* sp. (GenBank No. MK027124), respectively.

### Simultaneous Cr(VI) removal and pyrene degradation

**Figure 1:**
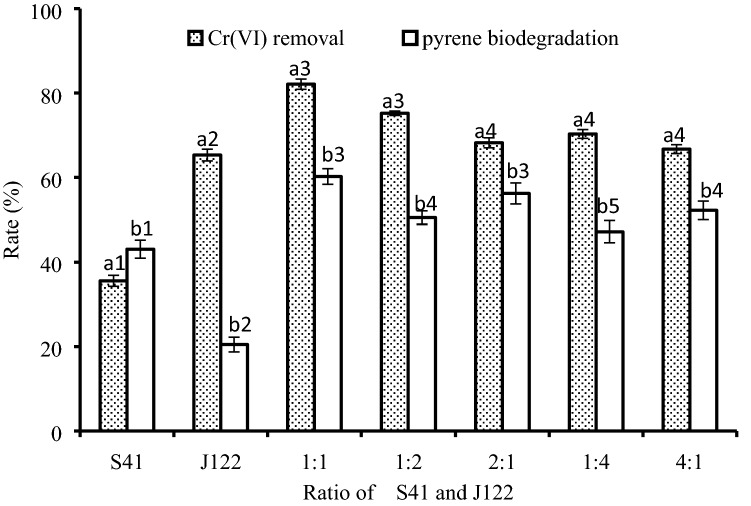
Percentage Cr(VI) removal and pyrene biodegradation by the isolated bacterial strains. S41 is strain S41, and J122 is strain J122. The original cultures of strains S41 or J122, or mixtures of the two strains with different ratios, were inoculated at 1% (% v/v, OD_600_ of 1.5) into sterile bottles containing MM and 30 mg/L Cr(VI) and 50 mg/L pyrene. The percentage Cr(VI)-removal was tested on day 3, and pyrene biodegradation was tested on day 7. The complete removal of 30 mg/L Cr(VI) within 3 days or complete degradation of 50 mg/L pyrene within 7 days was set to 100%. The error bars represent the standard deviations of triplicate samples. Same letter means one item; same letter and different numbers indicate significant (p < 0.05) differences of efficiencies of Cr removal/pyrene biodegradation among different groups.

Although both stains were able to remove Cr(VI) and biodegrade pyrene simultaneously, they had different optimal conditions for growth and biodegradation/reduction (Fig. 1). Strain S41 was more efficient at pyrene degradation than Cr(VI) removal, while strain J122 was more efficient at Cr(VI) removal than pyrene biodegradation. Therefore, a mixture of these strains was proposed to enhance the efficiency of Cr(VI) and pyrene removal. The simultaneous Cr(VI) removal and pyrene biodegradation by the strain mixture was better than for either strain alone; the highest efficiency was obtained for the strain mixture with 82.1 ± 1.22% removal of 30 mg/L Cr(VI) within 3 days and 60.2 ± 1.85% biodegradation of 50 mg/L pyrene within 7 days when both strains were mixed equally.

### Orthogonal test

The orthogonal experiment, L_9_(3^3^), was designed to determine the optimal temperature, pH and inoculation amount for the mixed inoculum. The highest efficiencies obtained were 48.43% removal of 30 mg/L Cr(VI) within 1 day, and 67.2% biodegradation of 50 mg/L pyrene over 7 days, which was obtained from the experimental combination A1B3C3, where A1 was temperature of 25 °C, B3 was pH 9.0, and C3 was inoculation amount of 15.0% (Table 1). The pH had the most influence on the efficiency of Cr(VI) removal (p < 0.05) and inoculation amount had the most influence on pyrene degradation when compared with pH and temperature.

**Table 1:** Results of orthogonal testing of biodegradation/removal capability of isolated strains.

Test no	A Temperature (℃)	B pH	C Inoculation amount (%)	Combination	Cr(VI)—removal rate (%, 1d)	Pyrene—biodegradation rate (%, 7d)
1	2	1	2	A2B1C2	8.73	15.60
2	1	2	2	A1B2C2	22.97	26.40
3	3	1	3	A3B1C3	12.97	19.60
4	1	1	1	A1B1C1	7.50	12.50
5	2	3	1	A2B3C1	25.37	38.50
6	3	3	2	A3B3C2	25.32	27.75
7	1	3	3	A1B3C3	48.43	67.10
8	2	2	3	A2B2C3	40.97	56.70
9	3	2	1	A3B2C1	2.03	6.50

A1, A2, and A3 were 25 °C, 30 °C, and 35 °C, respectively; B1, B2, and B3 were pH 7.0, 8.0, and 9.0, respectively; C1, C2, and C3 were 5.0%, 10.0%, and 15.0% (v/v) inoculum, respectively. The complete removal of 30 mg/L Cr(VI) within 3 days or complete degradation of 50 mg/L pyrene within 7 days was set to 100%.

### Hydroponic experiment

**Figure 2:**
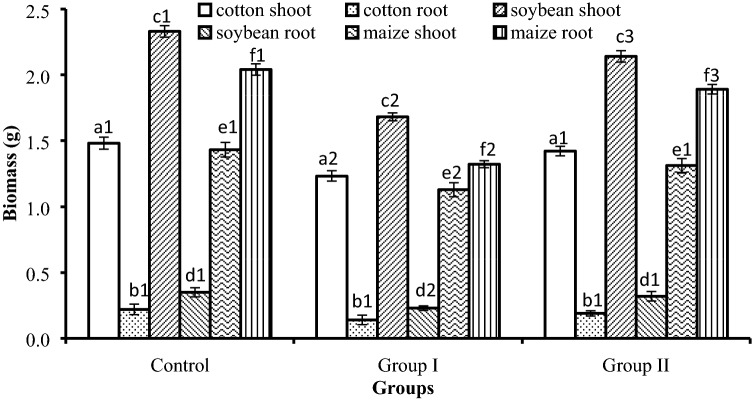
Biomass of cotton, soybean and maize in the hydroponic experiment. Control: plants were cultivated in nutrient solution without pyrene or Cr(VI) or mixed bacteria; shoot/root biomass of the control was set to 100%. Group I: plants were cultivated in nutrient solution with 20 mg/L pyrene and 20 mg/L Cr(VI). Group II: plants were cultivated in nutrient solution with 20 mg/L pyrene and 20 mg/L Cr(VI), and inoculated with strains S41 and J122 (1:1 ratio; total inoculation amount of 15% v/v). The error bars represent the standard deviations of 5 replicates. Same letter means one item; same letter and different numbers indicate significant (p < 0.05) differences of shoots/roots biomass among different groups.

Cotton, maize and soybean were negatively affected by exposure to pyrene and Cr(VI) for 10 days when compared with the control without pyrene or Cr(VI); the shoot and root biomass decreased by 16.9–36.4% (Fig. 2). When the mixed strains were added into group II with pyrene and Cr(VI), the shoot and root biomass mostly recovered and reached 86.4–95.9% of the original biomass in the control. For almost all items apart from cotton root, biomass was significantly different between group I where crops were exposed to pyrene-Cr(VI) and the control group without pyrene or Cr(VI) (p < 0.05); significant differences existed between group I and group II where crops were exposed to pyrene and Cr(VI) but were inoculated with the mixed bacteria. These results confirmed that pyrene and Cr(VI) inhibited plant biomass; the biomass with bacterial inoculation significantly increased in group II when compared with that in group I. For cotton shoot and soybean root, no significant difference was observed between the control and group II, which showed that the biomass almost completely recovered with bacterial inoculation. For soybean shoot, maize shoot, and maize root, significant differences were still observed between the control and group II, which suggested that the biomass partly recovered but still suffered some damage from exposure to the two contaminants.

After 10 days, pyrene and Cr(VI) concentrations were very low in experimental group II, reaching 2.10 ± 0.60 mg/L and 1.30 ± 0.44 mg/L, respectively, while they were 15.81 ± 1.72 mg/L and 18.09 ± 0.76 mg/L, respectively, in group I. Shoot/root biomass of the control 2, that plants were cultivated in nutrient solution with only two bacteria, had no significant differences with that of the control 1 that plants were cultivated in nutrient solution without bacteria (p < 0.05) (Supplementary Table 1), which suggested the bacteria had no influence on growth of the plants. The results indicated that bioremediation of the polluted water was mainly the result of bacterial inoculation; this was because pyrene and Cr(VI) were most efficiently removed from the hydroponic water in group II and the toxicity to these crops decreased greatly, which resulted in the effective recovery of shoot and root biomass.

## Discussion

*Serratia* sp. was previous identified as an effective Cr(VI)-reducer^16,17^, and was also able to degrade PAHs, including pyrene^18^ and BaP^19^. Cr(VI)-reduction and pyrene-degradation ability have been found in the genus *Arthrobacter*^10,20^. Previous studies showed that *Serratia* and *Arthrobacter* sp. could only reduce Cr(VI) alone or degrade pyrene alone. In this work, strains of these two genera were capable of simultaneous Cr(VI) removal and pyrene degradation.

Roots and shoots are important plant organs that can be negatively affected by contacting with heavy metals, such as Cr, cadmium and copper^7,21,22^. Zong et al.^7^ showed that there were positive correlations between total Cr in peanuts and total root length, root surface area, and root volume, and that Cr(VI) at concentrations ranging from 75 to 100 µmol/L significantly decreased the root and shoot biomass of peanut. Cr(VI) is a powerful oxidizing agent that can pass through cell membranes and undergo subsequent intracellular reduction to reactive intermediates^23^. Plant species can take up PAHs primarily through roots and translocate them to various aerial parts; the pyrene concentration in maize roots increased over time, while that in maize shoots decreased^24^. Mixed contamination with Cr(VI) and BaP was demonstrated to be more toxic than that of BaP alone on seed germination of *Lolium perenne*. BaP alone (at 1–4 mg/L) could accelerate the germination rate, while the joint toxicity of Cr(VI) and BaP significantly inhibited the germination rate^8^. Here, the joint toxicity of 20 mg/L pyrene and 20 mg/L Cr(VI) decreased the shoot and root biomass of cotton, maize and soybean, which showed that exposure to Cr(VI) and pyrene might be involved in changes in plant physiology and transport capacities.

Microbial consortia can be responsible for removing certain substances from water. Lee et al.^25^ showed that major nutrients (nitrogen and phosphorus) and inorganic cations (Ca^2+^, Mg^2+^, and Fe^2+^ ) were removed from a hydroponic system by a microbial consortium and microalgae, which they proposed was related to the potential of photosynthetic microbes for the treatment of waste nutrients. Other pathways and mechanisms, such as biosorption, biodegradation, and reduction, can be involved in pollutant removal. Pyrene catabolism is very difficult because of its complex four-ring structure. It has been hypothesized that most aromatic compounds are first converted to one of several di- or trihydroxylated substrates, such as catechol or proteocatechuate, whose aromatic ring can be enzymatically cleaved^26^. In bacteria, multiple genes of both catabolic and anabolic pathways are clustered together and co-regulated in operons, such as *ben* and *pca* cluster genes^27,28^, which are probably involved in pyrene biodegradation.The endophytic bacterium *Serratia* sp. PW7 successfully decreased pyrene accumulation and pyrene transportation from roots to shoots in wheat because of pyrene biodegradation by this strain^18^. Li et al.^29^ showed that *Mycobacterium* sp. strain A1-PYR degraded pyrene into metabolites that stimulated cell division of the green alga *Selenastrum capricornutum*; transcriptomic analysis showed that the bacterial pyrene metabolites substantially accelerated protein synthesis at the G1 phase of the algal cell cycle, which suggested a close relationship between bacterial pyrene transformation and the ecological effects of toxic contaminants. Cr(VI) reduction is usually the main pathway of Cr(VI) removal and detoxification process by direct or indirect mechanism^4,30^. In the direct mode, chromate reductases, such as ChrR, NfsA, and ChrT, are responsible for reducing Cr(VI) to Cr(III) in bacteria^16,31,32^, whereas reductants or oxidants detoxify Cr(VI) in indirect mechanism^4^. If Cr(VI) is reduced to Cr(III) extracellularly, this form of the metal is not readily transported into cells and so toxicity is not observed^23^. Microbes and plants sometimes become a symbiotic relationship which can help plants to become resistant to Cr(VI) toxicity. *Bacillus subtilus* MAI3 and PAW3 could produce antioxidants, also *B. subtilus* PAW3 generated substantial amounts of plant growth promoting substances, while they reduced Cr(VI)^33,34^. Both Cr(VI) reduction and improvement in cowpea or soybean growth were responsible for growth of plants under Cr(VI) stress. The two strains in this work had no such obvious influence on the growth of cotton, soybean and maize as demonstrated by the comparison of control 1 and control 2 in the hydroponic experiment. Because these crops were cultivated in nutrient solution in the control 1, while, cultured in nutrient solution with addition of the mixed strains in the control 2, and there is no significant difference of shoot/root biomass between the two groups. In the hydroponic culture of cotton, soybean and maize, the shoot and root biomass decreased when these crops were exposed to pyrene and Cr(VI), and then partly recovered because of decreased toxicity of pyrene and Cr(VI) because of pyrene biodegradation and Cr(VI) reduction by the mixed bacterial inoculant, not because of plant-growth-promoting function of the bacteria. The mixed inoculant showed potential for bioremediation of pyrene and Cr(VI). Due to toxicity of the pollutants and important use of crops, one of the future work will be that Cr(VI), pyrene and its transformed product benzo[a]pyrene (BaP) are further needed to detect in the plants tissues before use of the biomass in food and clothing etc., in order to prevent that probably Cr(VI) and/or BaP may have been translocated to the maize, soybean and cotton, and reach subsequently human beings.

## Materials and methods

### Chemicals, media and nutrient solution

Luria–Bertani (LB) medium was composed of 10.0 g/L tryptone, 5.0 g/L yeast extract, and 10 g/L NaCl; the pH was adjusted to 7.0 with NaOH. Mineral salt medium (MM) consisted of 1.0 g (NH4)_2_SO_4_, 0.2 g MgSO_4_·7H_2_O, 0.2 g CaCl_2_, 0.5 g NaH_2_PO_4_, 0.5 g K_2_HPO_4_, 0.2 g NaCl, and 0.01 g FeSO_4_ per liter, and the solid medium used to make agar plates was prepared as above with 1.5% agar powder. Knop solution was used as the nutrient solution to cultivate the crops^35^, and its composition was listed in Supplementary Table 2. All chemicals, including K_2_Cr_2_O_7_ and pyrene, were of at least analytical reagent grade. K_2_Cr_2_O_7_ solution with the concentration of 50 g/L was obtained via dissolving K_2_Cr_2_O_7_ in sterile water.

### Isolation of the bacterial strain and identification

Bacteria were isolated from soil and water samples that were collected from sites near electroplating facilities, tannery factories, garages and petrol stations in Wenzhou, China. Strains that could grow on MM agar plates containing 10 mg/L K_2_Cr_2_O_7_ and 50 mg/L pyrene were purified several times by streaking to single colonies on selective plates. Genomic DNA was extracted from two isolated bacterial strains; bacterial universal primers (5′-AGA GTT TGA TCC TGG TCA GAA CGC T-3′ and 5′-TAC GGC TAC CTT GTT ACG ACT TCA CCC C-3′) were used as primers to amplify 16S rRNA gene fragments from the isolated genomic DNA as previously described^36^. The 16S rRNA gene sequences were determined and the strains were identified through comparison of their 16S rRNA gene sequences with known database sequences accessed through the National Center for Biotechnology Information (https://www.ncbi.nlm.nih.gov/).

### Analysis of Cr(VI) removal and pyrene degradation

Strains were grown in LB liquid medium overnight until the optical density at 600 nm (OD_600_) was 1.5. An aliquot (0.5 mL) of the original culture was inoculated into 50 mL MM medium containing 30 mg/L K_2_Cr_2_O_7_, 50 mg/L pyrene and 50 g/L glucose as the carbon source for initiation of the experiment. The culture was aerobically incubated at 30 °C with shaking at 160 rpm for 7 days to determine the ability of the isolate to simultaneously remove Cr(VI) and biodegrade pyrene. An aliquot (1.5 mL) of the culture medium was withdrawn on day 3 and used to measure the residual Cr(VI) with the 1,5-diphenylcarbazide method as previously described^37^. After 7 days, the residual pyrene was extracted from each mixture; the extracted pyrene was analyzed by high performance liquid chromatography (HPLC, Waters 2690-5, USA) using a C18 Waters column (4.6 mm × 150 mm) with a mobile phase of 90% methyl alcohol and 10% water at a flow rate of 1.0 mL/min^37^.

### Determination of the ratio of two strains

Mixtures of the two strains with different ratios, containing 1:1, 1:2, 2:1, 1:4, and 4:1, were used to analyze the efficiencies of Cr(VI) removal and pyrene degradation. Then the best ratio was selected based on the highest efficiency.

### Orthogonal experimental design

Orthogonal testing is often used for optimization of methods^38,39^ because it is an efficient process when compared with traditional experimental design. Here, based on the results of single factor analysis (data not shown), the orthogonal experiment, L_9_(3^3^), involving three factors, namely, the temperature, pH and inoculation amount, was designed to determine the optimal conditions for pollutant removal by the mixed strains (Table 2).

**Table 2:** Factorial design of the orthogonal experiment.

Level	A Temperature (℃)	B pH	C Inoculation amount (%)
1	25	7.0	5.0
2	30	8.0	10.0
3	35	9.0	15.0

### Plant materials and pyrene: Cr(VI) exposure in hydroponic experiment

Cotton (*Gossypium* spp.), soybean (*Glycine max* (Linn.) Merr.) and maize (*Zea mays* ssp. mays L.) were used in this study. Seeds of each species were soaked in water for 24 h, and then pretreated with 30% H_2_O_2_ solution and rinsed with sterile water. Seeds that wrapped up by damp gauze were cultivated in petri dishes until their roots were about 2 cm long, and then the seeds without gauze were transferred and cultivated in damp sand. When the roots reached approximately 3 cm in length, the crops were transferred into the nutrient solution for the hydroponic experiment^40^. For each species, 30 seeds were cultivated, and almost all of them can grow roots, but their root lengths were a little different. Finally, 20 seeds of each species, having the roots with approximately 3 cm in length, were selected from that above 30 seeds, and used for the next hydroponic experiment.

Three groups of experiments were designed as follows. Experimental group I contained nutrient solution with Cr(VI) and pyrene at final concentrations of 20 mg/L of each. Experimental group II contained nutrient solution with 20 mg/L Cr(VI) and 20 mg/L pyrene; the nutrient solution was also inoculated with the mixed strains at 15% (% v/v; OD_600_ of 1.5). Experimental group III was the control, which contained nutrient solution alone. Another control group (named control 2) that plants were cultivated in the nutrient solution with only mixed strains was prepared for comparison. Each group was prepared with five replicates.

All groups were placed into the same incubator for 10 days. The plants were cultured under daytime conditions of 30,000 lx at 30 °C for 13 h, and nighttime conditions without light exposure at 25 ℃ for 11 h^23^. After 10 days of incubation, the biomass of roots and shoots, and the concentrations of pyrene and Cr(VI) in the nutrient solutions were analyzed.

### Statistical analysis

All statistical tests were performed using SPSS 20.0 software (p < 0.05). One-way analysis of variation (ANOVA) and Duncan multiple comparison were used to determine significant differences of shoot and root biomass among different groups in hydroponics, and also show differences of Cr-removal/pyrene-degradation efficiencies of mixed strains with different ratios.

## Supplementary Information

Supplementary Information.
