# Supplementary material for: Biotreatment of pyrene and Cr(VI) combined water pollution by mixed bacteria
Source: Sci Rep. 2021 Jan 8;11:114. doi: 10.1038/s41598-020-80053-2 (PMC7794335; doi:10.1038/s41598-020-80053-2)
Supplement: Supplementary file 1 — Supplementary Information. [file 41598_2020_80053_MOESM1_ESM.docx]

**Supplementary Table 1**  Shoot/root biomass of control 1 and control 2 in the hydroponic experiment

| Item | Biomass (g) | | Significant difference between control 1 and 2 |
| --- | --- | --- | --- |
|  | Control 1 | Control 2 |  |
| Cotton shoot | 1.494 ± 0.035 | 1.468 ± 0.043 | No |
| Cotton root | 0.222 ± 0.028 | 0.216 ± 0.036 | No |
| Soybean shoot | 2.368 ± 0.050 | 2.374 ± 0.043 | No |
| Soybean root | 0.338 ± 0.024 | 0.346 ± 0.030 | No |
| Maize shoot | 1.428 ± 0.023 | 1.422 ± 0.036 | No |
| Maize root | 2.032 ± 0.044 | 2.046 ± 0.040 | No |

Legend: Control 1: plants were cultivated in nutrient solution without pyrene or Cr(VI) or mixed bacteria. Control 2: plants were cultivated in nutrient solution with only two bacteria. ± means the standard deviation of 5 replicates.

**Supplementary Table 2** Composition of Knop solution

| Chemicals | Concentration (g/L) |
| --- | --- |
| Ca (NO_3_)_2_  KNO_3_  KH_2_PO_4_  MgSO_4_ • 7H_2_O  Solution I (EDTA-Fe)  Solution II (microelements) | 0.8  0.2  0.2  0.2  1ml /L  1ml /L |

Legend: Solution I was composed of 5.57 g FeS0_4_·7H_2_0 and 7.45 g Na_2_EDTA per liter. Solution II consisted of 2.86 g H_3_BO_3_, 1.81 g MnCl_2_, 0.08 g CuSO_4_·5H_2_O, 0.22 g ZnSO_4_·7H_2_O, and 0.09 g H_2_MoO_4_·H_2_O per liter.
